# Isoquercetin Improves Hepatic Lipid Accumulation by Activating AMPK Pathway and Suppressing TGF-β Signaling on an HFD-Induced Nonalcoholic Fatty Liver Disease Rat Model

**DOI:** 10.3390/ijms19124126

**Published:** 2018-12-19

**Authors:** Guohong Qin, Ji Ma, Qiongshan Huang, Hongli Yin, Jichun Han, Maoru Li, Yang Deng, Bing Wang, Waseem Hassan, Jing Shang

**Affiliations:** 1 School of Traditional Chinese Pharmacy, China Pharmaceutical University, Nanjing 211198, China; qinguo_0@163.com (G.Q.); matthewmj@163.com (J.M.); h569624132@163.com (Q.H.); yinhongli141516@163.com (H.Y.); hanjichun10@163.com (J.H.); limaoru2016@163.com (M.L.); youngd2018@163.com (Y.D.); weibowang2018@163.com (B.W.); 2 State Key Laboratory of Natural Medicines, China Pharmaceutical University, Nanjing 211198, China; 3 Jiangsu Key Laboratory of TCM Evaluation and Translational Research, China Pharmaceutical University, Nanjing 211198, China; 4 Department of Pharmacy, COMSATS University Islamabad, Lahore Campus, Lahore 54000, Pakistan

**Keywords:** isoquercetin, NAFLD, AMPK, TGF-β, Kupffer cell

## Abstract

Isoquercetin (IQ), a glucoside derivative of quercetin, has been reported to have beneficial effects in nonalcoholic fatty liver disease (NAFLD). In this study, we investigated the potential improvement of IQ in liver lipid accumulation, inflammation, oxidative condition, and activation in Kupffer cells (KCs) on a high-fat diet (HFD) induced NAFLD models. Male Sprague-Dawley (SD) rats were induced by HFD, lipopolysaccharides/free fatty acids (LPS/FFA) induced co-culture cells model between primary hepatocytes and Kupffer cells was used to test the effects and the underlying mechanism of IQ. Molecular docking was performed to predict the potential target of IQ. Significant effects of IQ were found on reduced lipid accumulation, inflammation, and oxidative stress. In addition, AMP-activated protein kinase (AMPK) pathway was activated by IQ, and is plays an important role in lipid regulation. Meanwhile, IQ reversed the increase of activated KCs which caused by lipid overload, and also suppression of Transforming growth factor beta (TGF-β) signaling by TGF-β Recptor-1 and SMAD2/3 signaling. Finally, TGF-βR1 and TGF-βR2 were both found may involve in the mechanism of IQ. IQ can improve hepatic lipid accumulation and decrease inflammation and oxidative stress by its activating AMPK pathway and suppressing TGF-β signaling to alleviate NAFLD.

## 1. Introduction

Nonalcoholic fatty liver disease (NAFLD) is a chronic liver disease with a global prevalence of 25%. In the general population, males are more likely to develop NAFLD than females. It is well-established that NAFLD is closely associated with type 2 diabetes, central obesity, dyslipidemia, and metabolic syndrome. In addition, some study about “lean NAFLD” has recently been reported that genetic factors may also related to the development and progression of NAFLD [1,2,3]. Rather than the “two-hit hypothesis”, NAFLD currently is considered as a systemic disorder with a complex multi-hit pathophysiology [4]. NAFLD has multiple stages from steatosis, histological phenotype nonalcoholic steatohepatitis (NASH) to advanced liver disease, cirrhosis, and hepatocellular carcinoma. Lipotoxicity, inflammation, innate immune activation, adipokines, microbiome, genetic, and environmental factors seem related to the complexity of NAFLD pathogenesis [5,6,7]. Upon this multi-hit pathophysiology, there are some different treatment options for the different stages of NAFLD, including reducing hepatic lipid accumulation at an early stage, preventing oxidative stress or inflammation at NASH, to reduce the risk of progressive fibrosis [8,9]. Diet control and physical exercise are the suggested treatment strategy.

So far, there is currently no licensed drug for the treatment of NAFLD, but some drugs, including antioxidant vitamin E, pioglitazone, and statins are under the clinical studies. However, due to comorbidities and potential side effects, neither drug has been widely adopted. There are also some other candidates currently undergoing clinical trials, such as obeticholic acid, cenicriviroc, and elafibranor [1,2].

AMP-activated protein kinase (AMPK) pathway is a master cellular energy metabolic switch reported to be involved in positive lipid regulation in the liver and is also well-established as the therapeutic target of NAFLD [10]. Recent studies showed that many compounds are able to activate AMPK in animal models and improve NAFLD [11]. Notably, the activity of AMPK can be reduced by pro-inflammatory cytokine [12,13]. During this, the anti-inflammation effect should be considered as a co-therapeutic strategy to improve liver inflammation of NAFLD when treated with AMPK activator.

Transforming growth factor beta (TGF-β), a pro-fibrosis cytokine in the liver, is secreted from activated Kupffer cells (KCs) [14,15]. TGF-β signaling has been proposed as a therapeutic target in the NAFLD patients and animal models [16,17]. TGF-β blocker was reported to alleviate NAFLD-associated fibrosis, but less improvement on hepatic lipid accumulation or steatosis [18]. Upon this, candidate compounds with multi-effects on lipid-lowering regulation, anti-inflammation, anti-oxidative stress, and anti-fibrosis seem to be a more suitable treatment strategy for NAFLD.

Isoquercetin (IQ) is a glucoside derivative of quercetin. Both IQ and quercetin can be found in many plants, such as *Cichorium glandulosum Boiss. et Huet* (*CG*), a traditional Chinese medicine that is present widely in the Aksu region of Xinjiang, China [19]. In addition to its robust anti-oxidant activity [19,20,21], various studies have highlighted the beneficial effects of IQ in NAFLD-related insulin resistance [22], fat oxidation [23], and hepatic steatosis [24] in both in vivo and in vitro models, as an anti-atherosclerotic [25] and anti-inflammatory agent [26].

Under the light shed by above-mentioned facts and growing recognition of NAFLD, this study seeks to demonstrate the potential use of IQ to treat hepatic lipid accumulation, inflammation, oxidative condition, and KCs activation on NAFLD by using the high-fat diet (HFD)-induced rat model.

## 2. Results

### 2.1. IQ Improves Lipid Accumulation on HFD Induced NAFLD Rats

**Figure 1:**
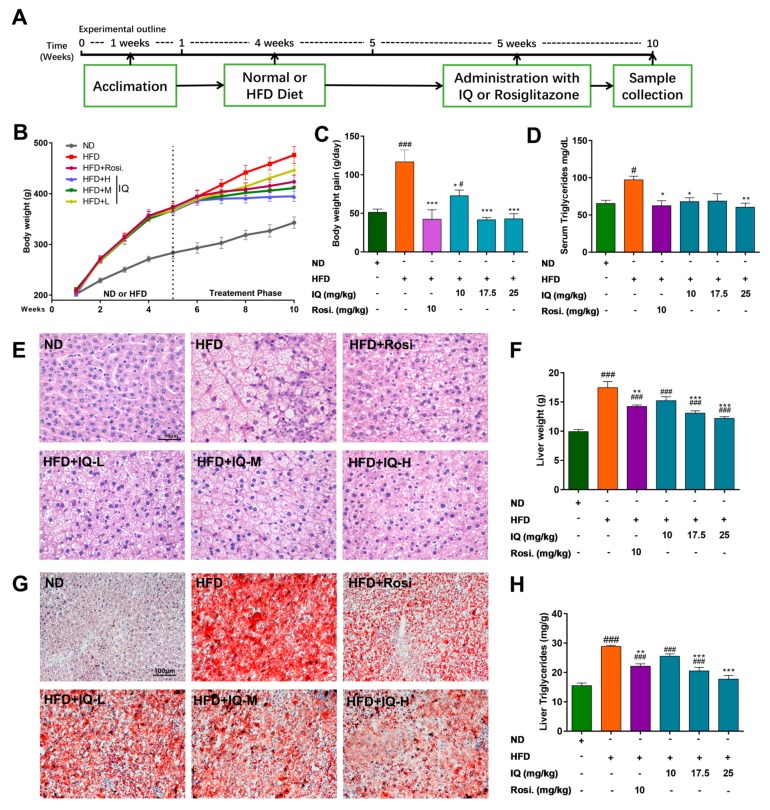
Isoquercetin (IQ) improves the condition of high-fat diet (HFD)-induced nonalcoholic fatty liver disease (NAFLD) rats by decreasing body weight and lipid accumulation. (**A**) The timelines and intragastrical administration schedule of various compounds used in the study; (**B**) the body weight curves of different groups used in the study; (**C**) the body weight gain/day during treatment intervention; (**D**) the serum triglyceride (TG); (**E**) demonstrated the haematoxylin and eosin stain (H&E) of the liver; (**F**) demonstrated the liver weight; (**G**) demonstrated the oil red stain of the liver; (**H**) illustrated the liver TG. The data represents ± SD. *p* < 0.05 was considered as statistically significant. # *p* < 0.05, ### *p* < 0.001 represent compared with normal diet (ND) group. * *p* < 0.05, ** *p* < 0.01, *** *p* < 0.001 represent compared with HFD group. The significant statistical difference as calculated by one-way ANOVA with Tukey’s post hoc test. (*n* = 6 each group).

In order to study the possibility of using IQ to treat NAFLD, we first established the rat model and then treated those rats with IQ. As shown in Figure 1A, the timelines and administration schedule of various compounds that were used in the study are explained. HFD induced NAFLD model [24,27] showed an obvious increase in body weight (Figure 1B), daily body weight gain during the treatment intervention (Figure 1C), and serum triglycerides (TG) (Figure 1D) when compared with a normal diet (ND). However, when those rats were treated with IQ, it appreciably reduced both raised body weight and serum TG in HFD-induced NAFLD rats. Histopathological analysis of liver tissue showed that rats fed with HFD alone developed a considerably high level of hepatic steatosis. Haematoxylin and eosin stain (H&E) staining features in liver sections (Figure 1E) showed a reversal of HFD induced steatosis with the intragastrical administration of IQ. Furthermore, this improvement was further confirmed by the fact that IQ also improved the HFD induced steatosis, as shown in oil red staining in a dose-dependent manner (Figure 1G). Similarly, liver weight (Figure 1F) and liver TG (Figure 1H) were notably ameliorated with the administration of IQ as compared to the HFD group, especially the middle dose treatment group (IQ-M, 17.5 mg/kg/day) and high dose treatment group (IQ-H, 25 mg/kg/day).

### 2.2. Effects of IQ on Oxidative Stress, Serum Cytokine Array, Inflammation and Kupffer Cell Markers

**Figure 2:**
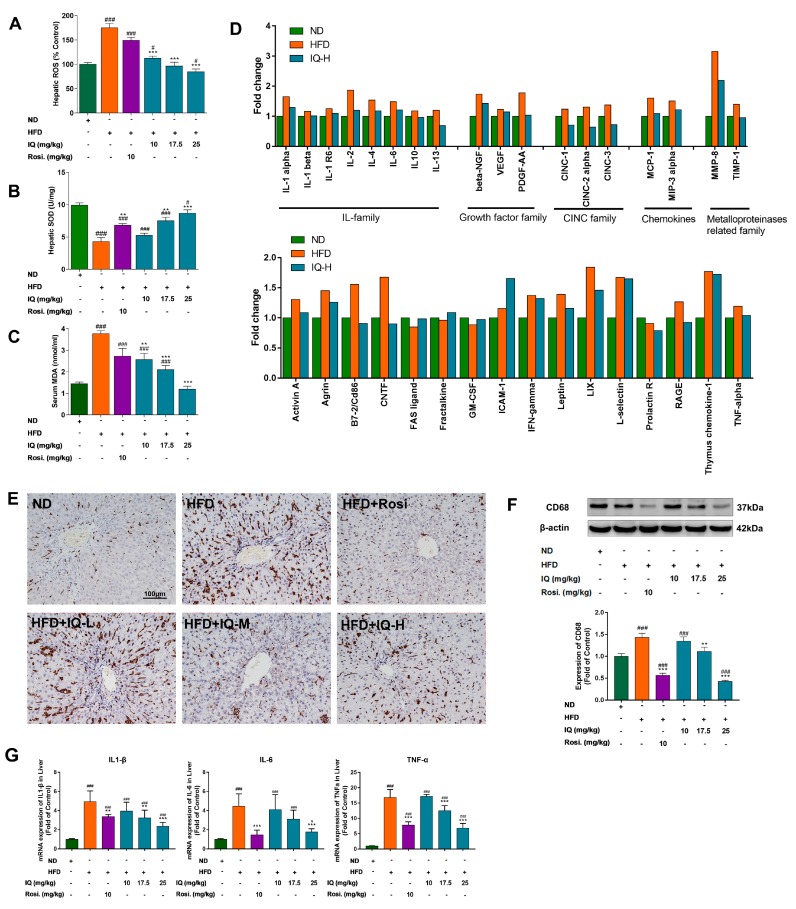
Effects of IQ on anti-oxidative stress, serum cytokine array, inflammation and regulation of Kupffer cell markers. (**A**) Illustrated the hepatic reactive oxygen species (ROS); (**B**) illustrated the hepatic superoxide dismutase (SOD); (**C**) illustrated the serum malondialdehyde (MDA); (**D**) the results of cytokine array, illustrated the graphical representation of interleukin (IL) family cytokine, cytokine-induced neutrophil chemoattractant (CINC) family, growth factors, chemokines, and matrix metalloproteinase (MMP) related cytokines; (**E**) immunohistochemical pictographs CD 68+ stain of liver; (**F**) western blotting analyses the changes of CD68 in liver; (**G**) mRNA expression levels of interleukin 1 β(*IL1 β*), *IL-6*, tumor necrosis factor-α (*TNF-α*) in the liver. The data represents ± SD. *p* < 0.05 was considered as statistically significant. # *p* < 0.05, ### *p* < 0.001 represent compared with the ND group. ** *p* < 0.01, *** *p* < 0.001 represent compared with HFD group. The significant statistical difference as calculated by one-way ANOVA with Tukey’s post hoc test. (*n* = 6 each group).

It is known that oxidative markers have significant changes in NAFLD patients, also as shown in the animal models. As mentioned-above, IQ has anti-oxidation activity, this effect was further tested in NAFLD rats, it is demonstrated that IQ can reverse these oxidative markers dose-dependently (Figure 2A–C).

In addition, serum cytokines and growth factors are the ultimate markers for many metabolic dysfunctions. Cytokine array was performed to find the gross changes in cytokine profile. As shown in Figure 2D, interleukin (IL) family (IL-1α, IL-2, IL-4, and IL-6), cytokine-induced neutrophil chemoattractant (CNIC) family (CNIC-1, CNIC-2 alpha, and CNIC-3), growth factor family, and MMP8 were different between HFD induced rats and ND group, while IQ-H showed partial probability to reverses these changes. Immunohistopathological photographs showed that CD68+ KC cells were increased in HFD induce rats. IQ was found to considerably normalize the CD68+ KC cells (Figure 2E). These results were further consolidated by western blot analysis, which also showed the suppression of CD68+ expression at middle and high doses of IQ (Figure 2F). The mRNA expression levels of *IL1 β*, *IL-6*, and tumor necrosis factor-α (*TNF-α*) in the liver were also reverse by IQ (Figure 2G).

### 2.3. IQ Enhances the AMPK Phosphorylation via LKβ1 Upstream

**Figure 3:**
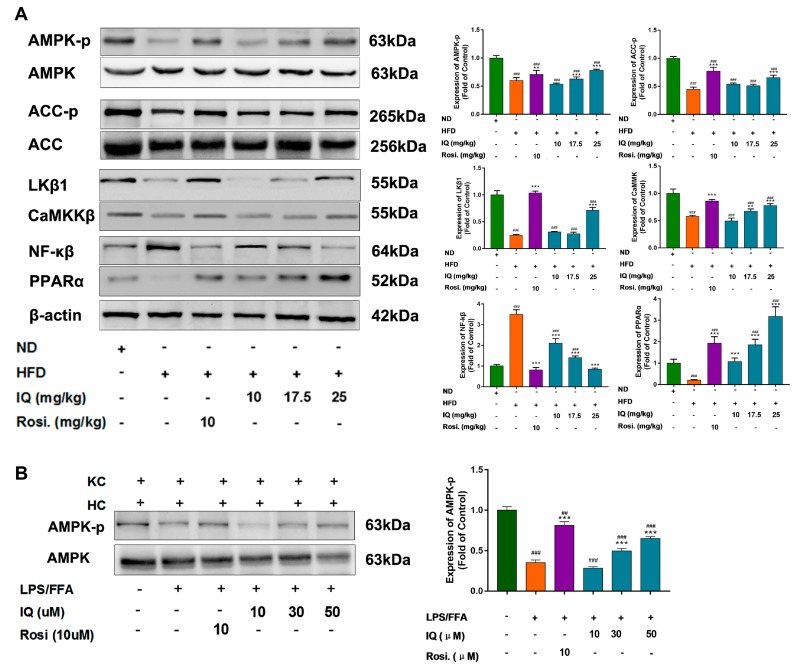
IQ enhances the AMP-activated protein kinase (AMPK) phosphorylation via Liver kinase β1 (LKβ1) upstream. (**A**) Western blot analysis of AMPK, phospho-AMPK (p-AMPK), Acetyl-CoA carboxylase (ACC), phospho-ACC (p-ACC), Liver kinase β1 (LKβ1), Calcium/calmodulin-dependent protein kinase kinase-1 (CaMKK1), Nuclear factor-κB (NF-κB), and Peroxisome proliferator-activated receptor alpha (PPAR-α); (**B**) representative blot for AMPK, p-AMPK in lipopolysaccharides/free fatty acids (LPS/FFA) induced in vitro co-culture model. ## *p* < 0.01, ### *p* < 0.001 represent compared with control. ** *p* < 0.01, *** *p* < 0.001 represent compared with model. The significant statistical difference as calculated by one-way ANOVA with Tukey’s post hoc test.

In order to trace the possible molecular mechanism that is responsible for lipid regulatory activities of IQ within hepatocytes, the AMPK pathway was particularly investigated. Interestingly, the high dose treatment group (IQ-H, 25 mg/kg/day) enhanced the phosphorylation of AMPK and Acetyl-CoA carboxylase (ACC) (Figure 3A) in HFD induced rats. Liver kinase β1 (LKβ1) and Calcium/calmodulin-dependent protein kinase kinase-1 (CaMKK1) are two major upstream regulators of AMPK. IQ dose proportionally upregulated LKβ1 protein expression (Figure 3A) in almost similar scheme as it phosphorylated AMPK. IQ-H significantly reversed HFD induced the downregulation of LKβ1 and CaMKK1. Further, peroxisome proliferator-activated receptor alpha (PPAR-α) and nuclear factor-κB (NF-κB) are also reported to be affected by IQ. Our data showed that protein expression of PPAR-α was enhanced and NF-κB has downregulated in IQ treated rats as compared to HFD fed rats (Figure 3A).

Furthermore, mRNA expression levels of downstream AMPK dependent lipogenic and lipolytic genes, such as fatty acid synthase (*FAS*), sterol regulatory element-binding transcription factor 1-C (*SREBP1-C*), *PPAR-γ*, and carnitine palmitoyltransferase 1 (*CPT-1*) were reversed by IQ in HFD induced rats. HFD fed group showed 6-, 3-, and 4-times increase in *FAS*, *SREBP1-C*, and *PPAR-γ* expressions, respectively, which were brought back to normal levels by IQ-H (Figure S1). *CPT-1*, fatty acid transporter to mitochondria, was also displayed an obvious increase in response to IQ (IQ-H) treatment (Figure S1). The ability of IQ to phosphorylate AMPK was reasserted in lipopolysaccharides/free fatty acids (LPS/FFA) induced co-culture model between hepatocytes and KCs. Treated with LPS/FFA decreases the phosphorylation of AMPK in the co-culture model, while the simultaneous delivery of IQ in the co-culture model upregulated the AMPK phosphorylation in middle and high dose (Figure 3B).

### 2.4. IQ Suppresses TGF-β Release, Downregulate TGF-βR1 in HFD Induced Rats and Co-Culture Model

**Figure 4:**
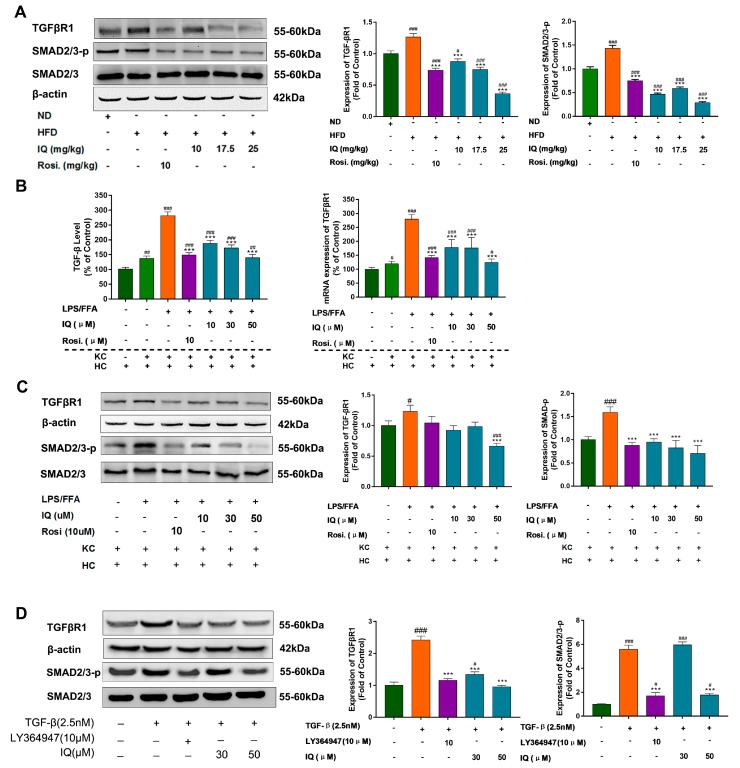
IQ suppresses TGF-β release, downregulate TGF-βR1 in HFD fed rats and co-culture model. (**A**) Representative blots of *TGF-βR1* and SMAD2/3 in HFD group; (**B**) shows the TGF-β level in LPS/FFA induced co-culture cell by ELISA test and the mRNA expression of *TGF-βR1* of LPS/FFA induced co-culture cell; (**C**) shows the representative blots of TGF-βR1 and SMAD2/3 in LPS/FFA induced co-culture model; (**D**) in vitro effects of IQ in TGF-β induced cultured cells (L02) on TGF-βR1 and SMAD2/3. # *p* < 0.05, ## *p* < 0.01, ### *p* < 0.001 represent compared with control. *** *p* < 0.001 represent compared with model. The significant statistical difference as calculated by one-way ANOVA with Tukey’s post hoc test.

IQ substantially suppressed TGF-βR1 and SMAD2/3 phosphorylation protein expression in comparison with HFD induced rats (Figure 4A). Additionally, co-culture between KC and hepatocytes showed elevated TGF-β release and *TGF-βR1* mRNA expression in the LPS/FFA induced model. Interestingly, IQ inhibits the expression of TGF-β and *TGF-βR1* in co-cultures (Figure 4B). Additionally, the downregulation of TGF-βR1 was confirmed, IQ also reduced the phosphorylation of the SMAD2/3, which is the downstream signal path of TGF-β (Figure 4C). To further consolidate the findings, the normal hepatocyte cell line L02 was utilized to be induced with recombinant TGF-β, while the effects of IQ were compared with the TGF-β receptor inhibitor (LY364947). Recombinant TGF-β substantially upgraded the expression of TGF-βR1 and raised the phosphorylation of downstream SMAD2/3 (Figure 4D). However, IQ and LY364947 reversed these changes, thus reaffirming the earlier findings.

### 2.5. Predict the Potential Mechanism of IQ by Molecular Docking

**Figure 5:**
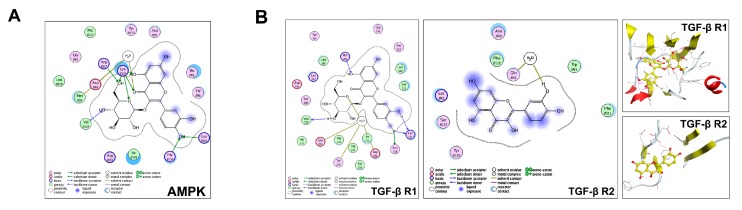
Predict the potential mechanism of IQ. (**A**) Docking results of IQ with AMPK. Amino acid residues which are thought to interact with IQ are shown in 2D representation using LigX in MOE (PDB number: 2YA3); (**B**) docking results of IQ with the TGF-β receptor, TGF-βR1 (access code: 1VJY.pdb), and TGF-βR2 (access code: 1KTZ.pdb) Amino acid residues, which are thought to interact with IQ are shown in two-dimensional (2D) representation using LigX in MOE. Hydrogen bonding is shown in dotted lines along with percentages.

To predict the possible target, molecular docking was performed to elucidate the nature of the association between IQ and AMPK or TGF-β receptors. The IQ structure (PDB code: 2YA3) displayed strong receptor contact with AMPK at thr B88, thr B86, ile B149, Arg B151, met B84 and Lys B126. 2YA3 also showed robust ligand affinity at –OH, and hydrogen binding sites (Figure 5A). This medicinal chemistry evidences confirmed the strong interaction and activity of IQ on AMPK. As is shown in Figure 5B, receptor contact was made between IQ and TGF-βR1 structure (Structure PDB code: 1VJY) at leu 340 and val 219. Similarly, the ligand was exposed to the receptor at 2 sites predominantly containing –OH, as measured and analyzed by MOE. The TGF-βR2 structure (Structure PDB code: 1KTZ) showed multiple strong receptor contact sites with different amino acids at ser B127, Try B 130, Asn B40, lys B42, and phe B111. The ligand was also exposed at multiple structural sites and with relatively stronger affinity than TGF-βR1. Molecular docking results confirmed the high degree of association between IQ and AMPK, TGF-βRs, revealed that AMPK, TGF-βR1, and TGF-βR2 have connected domain with IQ, which indicated the possible association with IQ.

## 3. Discussion

NAFLD has been considered as a systemic disorder with a complex multi-hit pathophysiology, so the simultaneous effect of multiple pathways may be a more effective research approach. In this study, we discovered the activities of IQ in the improvement of NAFLD and first revealed that effects of IQ are in the regulation of hepatic lipid accumulation, not only via AMPK pathway, but also partly via the TGF-β pathway, which is activated by KCs, and TGF-β is the key inflammatory cytokine that causes further fibrosis. Furthermore, it is predicted that the TGF-βR1 is the one but not the only potential target of IQ.

Our initial results indicate that the activated KCs, serum, and hepatic lipids were increased in the HFD-induced NAFLD model. Moreover, body and liver weights and hepatic oxidative stress were altered significantly in HFD fed animals as compared to the ND group. IQ significantly reduced HFD induced serum and hepatic lipid, body and liver weights, oxidative stress, and inflammation, pointing effect of IQ in hepatic lipid metabolism, anti-inflammation, and anti-oxidative stress. It is found that the activated KCs were pulled back by IQ in HFD induced group. Furthermore, serum cytokines array found that IQ can reverse the NAFLD, bringing the entire system to a normal state.

The AMPK pathway is a major metabolic switch that is reported to be involved in positive lipid regulation in liver [28,29]. Activation of AMPK requires phosphorylation on Thr-172, which is phosphorylated by both LKβ1 kinase and CaMKKβ (Ca^2+^/calmodulin-dependent protein kinase kinase β). More data recently illustrate that LKβ1 plays a crucial role in activating AMPK to control glucose and lipid metabolism in the liver [30]. CaMKKβ is viewed as an alternate upstream kinase that could also phosphorylate Thr-172 and activate AMPK in intact cells [31]. IQ-H significantly reversed the HFD induced LKβ1 and CaMKKβ downregulation, and then also enhanced the phosphorylation of AMPK. As being downstream of AMPK, ACC is an important rate-controlling enzyme for fatty acid synthesis and fatty acid oxidation in hepatocytes [32], and IQ can activate AMPK then increase phosphorylation of ACC, and reduce activity, thereby reducing fatty acid synthesis and increasing fat oxidation. Furthermore, SREBP-1c, FAS, and CPT-1 are linked with de novo lipogenesis [33], fatty acids synthesis [34], and the transportation of fatty acids to β-oxidation apparatus respectively. Moreover, AMPK and PPAR’s axis has a central role in lipid metabolism [35]. Importantly, AMPK has established itself as a master upstream regulator of all abovementioned metabolically vital genes [36]. The normalization of *FAS*, *CPT-1* and *SREBP-1-c*, *PPAR-γ*, and PPAR-α in IQ treated groups strengthened our hypothesis that AMPK is an upstream master regulator that is involved in lipid-lowering activities of IQ.

The KCs was highly involved in the progression of nonalcoholic fatty liver (NAFL) to NASH by the production of pro-inflammatory cytokines and oxidative stress. As shown in the HFD-induced rat model, activated KCs parallelly correlated with lipid accumulation in the liver [37]. TGF-β is one of the known KC secreted growth factor [38], and it regarded as the primary factor that drives fibrosis via the activation of SMAD2/3 signaling pathways [39,40]. Our previous observation has suggested that IQ was able to reduce systemic and local inflammation. Based on these facts and previous results, the effects of IQ in KC and TGF-β were analyzed by co-culture model between KCs and hepatocytes induced by LPS/FFA. As shown, IQ can decrease the expression of TGF-βR1 and the phosphorylation of SMAD2/3 in HFD induced rats and the co-culture model to inhibit the TGF-β pathway to alleviate fibrosis.

It is further confirmed that LPS/FFA induced co-culture of hepatocytes and KCs effectively secreted TGF-β as compared to hepatocytes alone. The suppression of TGF-β release by IQ strengthened our hypothesis that TGF-β secretion by KCs can be involved in the lipid modulation in hepatocytes. Co-culture experiments also showed lower *TGF-βR1* mRNA levels, which indicated that TGF-βR1 is a potential target of IQ. To further reveal the role of IQ, TGF-β receptor inhibitor was used to compare and confirm the reverse effect of IQ on present of recombinant TGF-β in vitro. Additionally, these in vitro findings in our HFD induced in vivo model were further confirmed by downregulation of TGF-βR1-SMAD2/3 signaling. Collectively, these results demonstrate the effects of IQ on TGF-βR1-SMAD2/3 signaling in lipid regulation, and TGF-βR1 is possibly a potential effect target of IQ.

Two well-known types of TGF-β receptor (TGF-βR1, TGF-βR2) exist on the cell surface. TGF-βR1 propagates the signal to the SMADs pathway, when it is activated by TGF-βR2. Using docking to reveal the possible role of TGF-βR1 and TGF-βR2 with IQ, it is predicted that IQ could connect with both TGF-βR1 and TGF-βR2, which can possibly explain that there are multiple targets that are connected with IQ i.e., TGF-βR1 and TGF-βR2 in TGF-β signaling.

To conclude, IQ can improve NAFLD by two pathways. One is the activation of AMPK pathway, and then regulates lipid accumulation and followed inflammation, oxidative stress. On the other hand, it can suppress TGF-β signaling by TGF-βR1-SMAD2/3, and then alleviate fibrosis.

However, less is known regard how to link these two pathways, it would be meaningful to study the relationship between AMPK and the TGF-β pathway in the future.

## 4. Materials and Methods

### 4.1. Animal Model and Experimental Design

IQ was purchased from Chengdu PushBio technology Co., Ltd. (Chengdu, China). The purity of the compound was 99%. 36 male Sprague-Dawley (SD) rats (180 to 200 g) were purchased from Qinglongshan Animal Laboratory Center (Shanghai, China) and was kept at controlled conditions of temperature (22 ± 1 °C) and humidity (60% ± 10%), and exposed to 12 h of light/dark cycle during the study. After the acclimatization period, rats were randomly divided into six groups (*n* = 6), as follows: (1) normal diet (ND) group, fed with normal diet and carboxymethylcellulose (CMC-Na+) (Sinopharm Chemical Reagent Company, Shanghai, China); (2) HFD group, supplemented with high caloric diet model; (3) IQ high dose treatment group (IQ-H), received HFD and IQ (25 mg/kg/day); (4) IQ middle dose treatment group (IQ-M), was given HFD and IQ (17.5 mg/kg/day); (5) IQ low dose treatment group (IQ-L), fed with HFD and IQ (10 mg/kg/day); and, (6) Rosiglitazone (Sigma-Aldrich, California, USA) treatment group (Rosi), administered HFD and Rosi (10 mg/kg/day). All groups received their respective treatments for four weeks HFD, and five weeks HFD with intragastrical administration of IQ and Rosi. The IQ and Rosi were suspended in a 0.5% carboxymethylcellulose solution. Normal diet (ND) and HFD (Table 1) was provided by Jiangsu Xietong Medical and Biological Corporation (Nanjing, China). The treatment schedule is illustrated in Figure 1A. The study was approved by the Ethical Committee of China Pharmaceutical University, Nanjing, and Laboratory Animal Management Committee of Jiangsu Province (SYXK (SU) 2016-009, 4 May 2016). 

### 4.2. Physical and Biochemical Analysis

After the completion of study duration, final body weights were analyzed before all rats were sacrificed by carbon dioxide asphyxiation and blood was withdrawn instantaneously by cardiac puncture. Serum and liver samples were collected and stored for further analysis. Serum and hepatic fat levels of total triglycerides (TG) were investigated by commercially available kits (BHKT, Beijing, China). Serum and hepatic quantities of superoxide dismutase (SOD) and malondialdehyde (MDA) were analyzed by commercial kits from Nanjing Jiancheng Bioengineering Institute (Nanjing, China).

### 4.3. Immunohistopathological Analysis

Histopathological analysis was performed by incising standardized specimen from the liver. Liver tissues were fixed in 10% formalin and processed for hematoxylin-eosin (H&E) staining as standard procedure. To evaluate fat deposition, liver sections were stained with oil red O (Sigma-Aldrich, St. Louis, MO, USA), according to standard protocols [41]. KCs were immune-stained with a monoclonal mouse anti-rat CD68 (1:800; Abcam, Cambridge, UK) antibody. The reaction was visualized under a light microscope (Olympus-BX53) by using biotin-conjugated secondary antibody.

### 4.4. Measurement of ROS

Intracellular reactive oxygen species (ROS) was measured by Reactive Oxygen Species Assay Kit (BeyoTime, Haimen, China) using 2, 7-dichlorofluorescein diacetate (DCF-DA), as mentioned elsewhere [42]. The production of DCF was analyzed at an excitation wavelength of 488 nm and an emission wavelength of 510 nm for 10 min by using fluorescence spectrometer.

### 4.5. Cytokine Array

Serum cytokines were analyzed by using rat cytokine antibody array (RayBiotech, Inc., Norcross, GA, USA), as described. Briefly, array membranes were blocked before the samples were incubated at 4 °C overnight. Membranes were washed and incubated with diluted biotin-conjugated primary antibodies cocktail (1:250) overnight, followed by incubation with diluted horseradish peroxidase-conjugated streptavidin (1:1000) at RT for 2 h. Membranes were detected by using X-ray film (Kodak X-OMAT AR film) after peroxidase substrate (detection buffers C and D; RayBiotech, Inc., Norcross, GA, USA) was exposed to membranes for 5 min in the dark. Signal intensities were obtained by Bio-Rad Imaging System and were analyzed with Quantity One software (Bio-Rad, Hercules, CA, USA).

### 4.6. Western Blotting

The proteins suspension of liver tissues were obtained using a total protein extraction kit (APPLYGEN, Beijing, China) and protein concentrations were determined by the BCA protein assay kit while using BSA as a standard. Subsequently, standard western blot procedures were followed, as described elsewhere [41]. β-actin was used as an endogenous control and blots were quantified by using Image J Software (NIH, Bethesda, MD, USA). The primary and secondary antibodies used for western blot (AMPK, AMPK-p, p-ACC, ACC, CD68, and β-actin) were purchased from Abcam (Cambridge, UK) and other antibodies (CaMKKβ, NF-κβ, TGF-βR1, PPARα, SMAD2/3-P, and SMAD2/3) were obtained from Santa Cruz Biotechnology (Santa Cruz, CA, USA).

### 4.7. Isolation of Primary Hepatocytes and Kupffer Cells

Isolation of rat hepatocytes was performed, as mentioned elsewhere [43], with some modification. Shortly, liver of rats that was anesthetized by sodiumpentobarbital (70 mg/kg) was perfused with perfusion buffer-1 (EDTA, 0.5 mM; HEPES, 25 mM, prepared in Hanks balance salt solution without Ca^2+^, Mg^2+^) and perfusion buffer-2 (Collagenase-II) at a constant speed. After the meshy appearance, Livers were excised, shredded, and filtered, followed by centrifugation at 50 g for 3 min at 4 °C. The pellets containing hepatocytes were further purified by using 90% percoll gradient. Counted hepatocytes were identified (CK 18 antibody) and cultured in well-plates.

KCs were isolated from normal rats as mentioned elsewhere [44] with some modification. Briefly, supernatants obtained after centrifugation at 50 g for 3 min were further centrifuged at 450× *g* for 10 min at 4 °C to sediment non-parenchymal cells. Pellets were collected and two-step percoll (Biosharp, Hefei, China) gradient cushion 25%/50% (*v*/*v*) was used to centrifuge cells at 1000× *g* for 10 min at 4 °C. A layer between 25% and 50% percoll cushion was collected and plated into culture wells after counting.

### 4.8. Co-Culture Model

KC’s and hepatocytes were co-cultured at approximately 1:4 ratios in six-well cell culture inserts (Millipore; cat. no. PIHT30R48). Cells were co-cultured in Williams E (Invitrogen, Carlsbad, CA, USA) medium, supplemented with 100 unit/mL penicillin and 100 mg/mL streptomycin. Lipopolysaccharides (LPS) (Sigma-Aldrich, St. Louis, MO, USA) (10 ng/mL) and free fatty acids (FFA) (Aladdin, Shanghai, China) (0.5 mM) prepared in 1% bovine serum albumin (BSA) (Yeason Biotech, Shanghai, China) were used to induce KCs, hepatocytes were cotreated with IQ in co-culture model at the same time. After 24h, the cells were collected to detect [45].

### 4.9. Cell Culture

L02 cells were obtained from the Cell Bank of the Chinese Academy of Science and were cultured in DMEM high-glucose medium (Hyclone, Logan, UT, USA), supplemented with 100 unit/mL penicillin, 100 unit/mL streptomycin, and 10% (*v*/*v*) fetal bovine serum (Hyclone, Logan, UT, USA). Cells were cultured in six-well-plates for experiments. L02 cells were treated with recombinant TGF-β (Bioscience, Shanghai, China), LY364947 (Tocris, Ellisville, MO, USA), and IQ at specified doses in DMEM without FBS.

### 4.10. ELISA

TGF-β levels in hepatocytes and co-culture model were measured by an enzyme-linked immunosorbent assay (ELISA) kit (ebioscience, San Diego, CA, USA), according to manufacturer’s instruction.

### 4.11. Quantitative Real-Time PCR

Total RNA was extracted from cells or liver tissues using TRIZOL reagent (Gibco-BRL, Waltham, MA, USA). cDNA was synthesized with PrimeScript™ RT Master Mix (Takara, Shiga, Japan), according to the manufacturer’s instructions. The PCR was performed on an iQ5 multicolor real-time PCR detection system (Bio-Rad, Hercules, CA, USA) by using SYBR^®^ Premix Ex TaqTM2 (Takara, Japan), as specified in manuals. Primer sequences are shown in (Table 2). The expression level of each gene was normalized to GAPDH.

### 4.12. Statistical Analysis

Statistical analysis was performed by using graph pad prism Version 5.0c (GraphPad Software, San Diego, CA, USA). Quantitative data are expressed as mean ± SD. Data were analyzed by one-way ANOVA with Tukey’s post hoc test. *p* < 0.05 was accepted as statistically significant.

## Figures and Tables

**Table 1:** The composition of experimental diets.

Ingredients	HFD (g/100g)
lard	10
bile salt	0.5
cholesterol	1
powdered milk	5
egg yolk powder	3
sugar	5
methylthiouracil	0.2
basal diet *	75.3

* Basal diet contains the following (g/100g): 13.3 fats, 26.2 proteins, and 60.5 carbohydrates.

**Table 2:** Primer sequences used in the study.

Gene	Species	Forward (F) and Reverse (R) Primer Sequence
*SREBP-1C*	rat	F: ACAGCACAGCA ACCAG AAACTC R: TTCATGCCCTCCATAGACACAT
*FAS*	rat	F: TTGGCTTAG TGAT TGCATCTCGT R: CAGGGTCTCTGTCCTCCTTTTGT
*PPAR-γ*	rat	F: GAAGCCCTTTGGTGACTTTATG R: AGGTTGTCTTGGATGTCCTCGA
*CPT-1*	rat	F: TCAGAGGATGGACACTGTAAAGGAG R: CCGAAAGAGTCAAATGGGAAGG
*TGF-βR1*	rat	F: CCACAGAGTAGGCACTAAAAGGTAT R: ACAAGATCATAGTAAGGCAACTGGT
*TNF-α*	rat	F: CACCATGAGCACGGAAAGCATGA R: CGCCTCACAGAGCAATGACTCCA
*IL-6*	rat	F: CACTTCACAAGTCGGAGGCT R: AGCACACTAGGTTTGCCGAG
*IL1 β*	rat	F: AGGAGAGACAAGCAACGACA R: CTTTTCCATCTTCTTCTTTGGGTAT
*GAPDH*	rat	F: CAACGGGAAACCCATCACCA R: ACGCCAGTAGACTCCACGACAT

## Supplementary Materials

Supplementary materials can be found at http://www.mdpi.com/1422-0067/19/12/4126/s1.
