# Supplementary material for: Isoquercetin Improves Hepatic Lipid Accumulation by Activating AMPK Pathway and Suppressing TGF-β Signaling on an HFD-Induced Nonalcoholic Fatty Liver Disease Rat Model
Source: Int J Mol Sci. 2018 Dec 19;19(12):4126. doi: 10.3390/ijms19124126 (PMC6321444; doi:10.3390/ijms19124126)
Supplement: Supplementary file 1 [file ijms-19-04126-s001.zip › ijms-394936-supplementary-final/ijms-394936-Supplementary Materials.pdf]

Supplementary materials

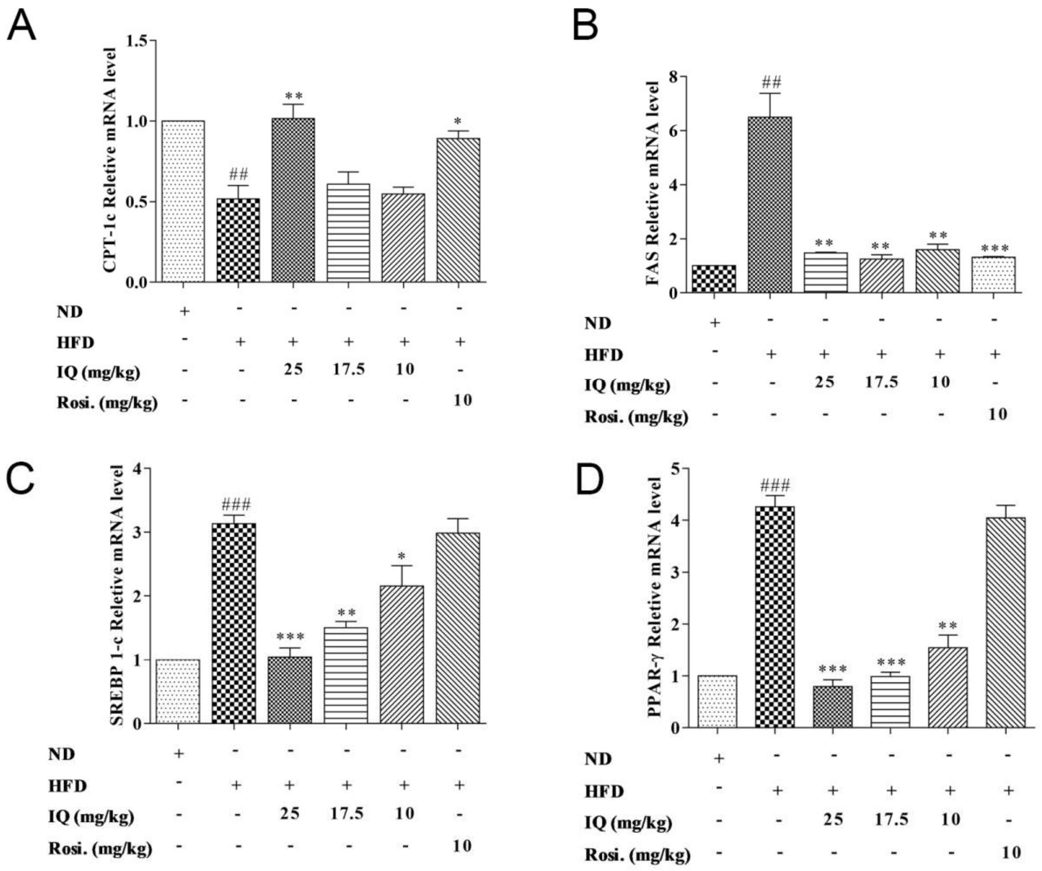

Figure S1. IQ regulated lipogenic and lipolytic genes on HFD induced NAFLD rat. (A) CPT-1. (B) FAS. (C) SREBP1-C. (D) PPAR-γ. The data represents ± SD. P<0.05 was considered as statistically significant. ##P<0.01, ### P<0.001 represent compared with control. * P<0.05, ** P<0.01, *** P<0.001 represent compared with model. The significant statistical difference as calculated by ANOVA followed by turkey's test. (n=6 each group).
